# Haemoglobin oxygen affinity in patients with severe COVID‐19 infection

**DOI:** 10.1111/bjh.16888

**Published:** 2020-06-28

**Authors:** Yvonne Daniel, Beverley J. Hunt, Andrew Retter, Katherine Henderson, Sarah Wilson, Claire C. Sharpe, Michael J. Shattock

**Affiliations:** ^1^ Viapath Blood Sciences Laboratories Guy's & St Thomas’ Hospital London UK; ^2^ Department of Haematology Guy's & St Thomas' NHS Foundation Trust London UK; ^3^ Intensive Care Guy's & St Thomas' NHS Foundation Trust London UK; ^4^ Emergency Medicine Guy's & St Thomas' NHS Foundation Trust London UK; ^5^ Department of Renal Sciences King's College London London UK; ^6^ School of Cardiovascular Medicine and Sciences King's College London London UK

**Keywords:** COVID-19, haemoglobin, hypoxaemia, oxygen affinity, oxygen dissociation, oxygen association

Severely ill COVID‐19 patients have an atypical form of respiratory distress. Acute respiratory distress syndrome (ARDS), while heterogenous, classically presents with severe hypoxia and decreased lung compliance.^1^ In SARS‐CoV‐2 coronavirus infection (COVID‐19) lung mechanics and compliance are relatively well conserved until late in the disease course. Despite early preservation of lung compliance, the hypoxaemia in COVID‐19 is severe and is ultimately the primary mechanism of multiple organ failure and death.^2^ The underlying pathology is due to COVID‐19 entering cells via the ACE2 receptor. This receptor is expressed on many cells including alveolar epithelial cells and vascular endothelium resulting in a profound immune response and widespread endothelial dysfunction.^3^

We were both interested and concerned to read the report by Liu and Li.^4^ This *in silico* study used homology modelling and molecular docking algorithms to predict theoretical interactions between COVID‐19 and haemoglobin (Hb). COVID‐19 expresses a variety of open reading frame proteins including orf1ab, ORF3a, ORF6, ORF7a, ORF10 and ORF8. Liu and Li^4^ predict that these ORF proteins can interact with haemoglobin to reduce both oxygen (O_2_) affinity and total haemoglobin content. They report specifically that their modelling predicts a theoretical interaction between orf1ab, ORF10 and ORF3a and the haem moiety of the beta chain while ORF8 and viral surface glycoproteins can directly target the porphyrin. They assert that their data support the use of chloroquine and hydroxychloroquine as therapeutic agents. However, the methods used by Lui and Li have very recently been criticised — not least of all because they are unsupported by experimental evidence.^5^ It is unclear what testable physiological consequences might arise from an interaction between viral proteins and Hb but these are presumed to include a generalised loss of Hb and/or a change in O_2_ affinity. While mild anaemia and decreased Hb content have been reported,^6^ ^,^ ^7^ to our knowledge the effect of COVID‐19 on O_2_ affinity has not been investigated.

Multiple studies and national guidelines strongly support restrictive transfusion practice in the critically ill as a basic standard of care.^8^ It is possible that if the haemoglobin molecule was adversely affected by COVID‐19, and the O_2_ affinity curve right‐shifted, restrictive transfusion could be potentially harmful. Given the very high mortality of these ventilated COVID‐19 patients, we considered evaluation of any effect on the O_2_ affinity curve as urgent to inform our practice.

Physicians caring for patients infected with COVID‐19 ordered an Hb O_2_ affinity assessment in 14 patients (four newly admitted from the emergency department and 10 patients receiving mechanical ventilation in critical care) which we compared to 11 age‐ and sex‐matched controls as part of an audit to assess the utility of this investigation in the management of patients with severe disease (Guys and St Thomas’ Hospital Audit No. 10855; Table I). No extra samples or blood tests were taken from patients — rather relevant samples were identified after a routine full blood count had been reported.

**Table I:** Patient characteristics and measurements of haemoglobin, ferritin, CRP and O_2_ affinity. Data are mean ± *SD* (*n*). All control data came from patients whose samples were already analysed for a full blood count and had shown normal red cell indices and normal Hb variants by high performance liquid chromatography (HPLC). No extra samples were taken. Statistical analyses (*P* values) were derived from a standard Student’s *t*‐test and differences were considered significant at *P < 0·05). ns = not significant. na = not available.

	Control	COVID‐19	P
Age	50·8 ± 10·3 (11)	58·6 ± 16·3 (14)	ns (0·18)
Sex (male:female)	5 (45%):6 (55%)	7 (50%):7 (50%)	–
Total Hb (g/l)	143 ± 11 (11)	93 ± 23 (14)	*(<0·0001)
HbA2 (%)	2·9 ± 0·1 (5)	2·8 ± 0·3 (14)	ns (0·22)
PCV	0·43 (11)	0·29 ± 0·06 (14)	*(<0·0001)
Ferritin (µg/l)	na	1,208 ± 1,328 (13)	–
CRP (mg/l)	9 ± 10 (4)	155 ± 125 (12)	* (<0·02)
O_2_ affinity P50 (mm Hg)	28·5 ± 1·8 (11)	29·0 ± 2·3 (14)	ns (0·40)
O_2_ affinity Hill Slope (x10^−2^)	2·75 ± 0·15 (11)	2·72 ± 0·09 (14)	ns (0·53)

O_2_ saturation curves from control and COVID‐19‐positive patients are shown in Fig 1. It is clear that there are no differences in O_2_ affinity or co‐operativity between samples from COVID‐19 and matched controls. The P50 and Hill Slope values are given in Table I. However, as is common in critically ill patients, there is a substantial anaemia and loss of total haemoglobin. In summary, while the total O_2_‐carrying capacity will certainly be reduced by the anaemia and loss of total haemoglobin, we see no evidence, using standard clinical measures in a small cohort of patients, to suggest that COVID‐19 alters haemoglobin O_2_ affinity and there is no requirement to change our transfusion practice.

**Fig 1:**
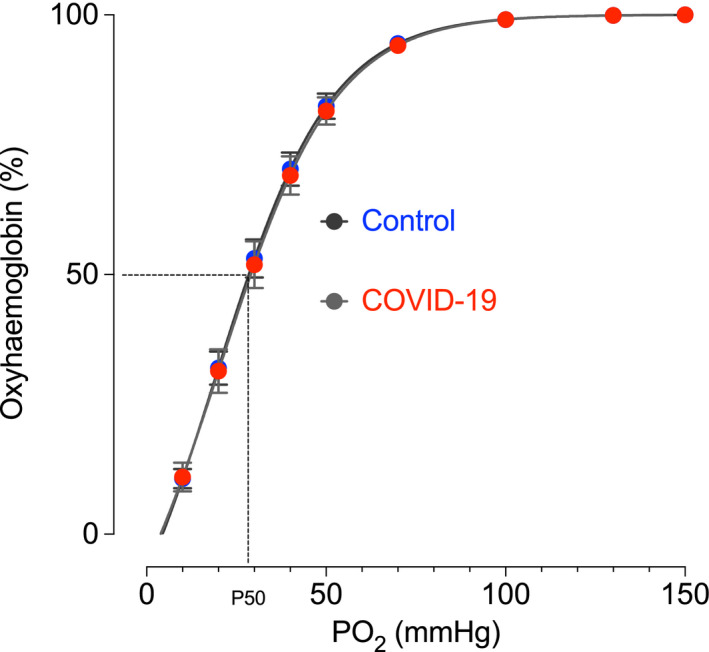
Haemoglobin O_2_ affinity curves from patients testing positive for COVID‐19 and age‐ and sex‐matched controls. Measurements were made using a Hemox Analyzer (TCS Scientific, New Hope, PA, USA) at pH 7·4 and 37°C. Each dataset from each sample was fitted using a sigmoidal function with variable Hill Slope according to the equation O_2_ Sat (%) = Bottom + [(Top − Bottom)/1 + 10^(LogEC50 − PO_2_) * HillSlope] where the 'Top' was constrained to 100%. The data points shown, and the P50 and Hill Slope values in Table I, were derived from each sample, and each fitted curve, and averaged to give a mean ± *SD* [*n* = 14 (COVID‐19 positive), *n* = 11 (Control)]. The curves shown are fitted to the average data points using the same equation. Confidence intervals are small and overlapping (not shown).

## Author contributions

CCS and MJS conceived and designed the study; BJH provided access to Viapath analytical labs and haematology expertise; RA and HK were responsible for patient care; YD and SW analysed blood samples and patient data; MJS analysed the data and wrote the first draft; all authors revised and approved the final submission.
